# Primary Lung Cancer Organoids for Personalized Medicine—Are They Ready for Clinical Use?

**DOI:** 10.3390/cancers13194832

**Published:** 2021-09-28

**Authors:** Raphael S. Werner, Michaela B. Kirschner, Isabelle Opitz

**Affiliations:** Department of Thoracic Surgery, University Hospital Zurich, 8091 Zurich, Switzerland; raphael.werner@usz.ch (R.S.W.); michaela.kirschner@usz.ch (M.B.K.)

**Keywords:** non-small cell lung cancer, organoids, tumoroids, cancer model, personalized medicine

## Abstract

**Simple Summary:**

Identification of novel therapeutic strategies for the treatment of non-small cell lung cancer (NSCLC) remains an urgent need in the area of lung cancer research. Traditional in vitro cell culture systems form one of the corner stones of cancer research. However, the growth of cancer cells in a two-dimensional pattern on a plastic dish in artificial conditions is not ideal, as the growth pattern significantly differs from that of a complex tumor in a patient. Hence, in recent years, efforts have been undertaken to generate more complex three-dimensional in vitro models, so-called organoid or tumoroid models, that more closely resemble the growth pattern and tumor heterogeneity observed in a patient. In this review, we summarize the efforts thus far undertaken in the area of NSCLC organoid development.

**Abstract:**

Despite many developments in recent years, non-small cell lung cancer (NSCLC) remains the leading cause of cancer-related death worldwide. Therefore, additional research, aiming to further elucidate the underlying molecular mechanisms of malignant transformation and development of therapy resistance, as well as the identification of additional novel therapeutic avenues, is crucial. For this purpose, reliable in vitro models are indispensable, as they allow for quick identification of suspected oncogenic drivers or evaluation of novel therapeutic strategies in a timely and cost-effective fashion. However, standard two-dimensional cell culture systems, the most frequently used in vitro model, are usually not truly representative of the situation in a patient as these models lack the tumor heterogeneity, the surrounding tumor microenvironment and the three-dimensional complexity of a tumor in vitro. For this reason, 3D cell culture systems, in particular organoids generated from normal non-malignant cells or tumor cell-based organoids (tumoroids), have in recent years gained much attention as alternative in vitro model systems that more closely resemble the actual primary tumor. In this review, we provide an overview of the available literature in the field of NSCLC organoids, which might still be in its infancy, but is gaining momentum.

## 1. Introduction

Lung cancer to date is the leading cause of cancer-related death worldwide, accounting for approximately 1.8 million deaths (18%), although in 2020 it was “only” the second most commonly diagnosed cancer (11.4%), having been marginally overtaken by breast cancer (11.7%) [1].

Non-small cell lung cancer (NSCLC) accounts for approximately 85% of all lung cancer cases. In addition to the histopathological subclassification into adenocarcinomas (AC), squamous cell carcinomas (SCC) and large cell neuroendocrine carcinomas (LCNE), further subdivision based on molecular characteristics in recent years has been shown to be of additional importance as a number of the known oncogenic driver mutations in lung cancer are targetable. Unfortunately, in a significant number of patients with NSCLC, no targeted treatment options are available to date. A targeted treatment is only available in approximately 80% of all patients with lung AC and most commonly includes a treatment of sensitizing alterations in EGFR, ALK, ROS1, RET, MET or BRAF. Although 50% of SCCs present with an amplification in FGFR1 or an aberration in PI3K, neither are clinically targetable and the frequency of targetable driver mutations is so low that molecular testing is not routinely performed [2]. Consequently, it remains an urgent clinical need to identify additional therapeutic targets for those patients currently having to rely on standard chemotherapy but failing to respond, as well as second-line options for any targeted therapy patient developing resistance mechanisms.

While molecular characterization of the primary tumor is certainly crucial for the identification of potential novel therapeutic targets, it is equally important to be able to comprehensively evaluate any new therapeutic strategy in preclinical models. The first choice for the evaluation of novel therapeutic components is usually established commercially available cell lines grown in 2D. These are of course easily accessible and straightforward to handle, but also fundamentally flawed as they do not reflect and recapitulate the complex tumor heterogeneity of the primary tumor within the patient that will later be treated with the respective drugs. The complex composition of heterogenous tumor cells with different molecular phenotypes, together with the surrounding tumor microenvironment, is, however, what ultimately determines if and how a tumor responds to a given treatment. Application of complex, multicellular, three-dimensional organoid systems instead of standard 2D cell culture systems is therefore likely to provide a better assessment of the usefulness of a certain drug for the treatment of NSCLC in an early stage of preclinical assessment, and might even be able to avoid at least in part some of the preclinical animal testing.

## 2. Lung Cancer Organoid Methodology

The establishment of human epithelial airway organoids derived from nasal polyps and cultured within collagen lattices was first described in 1993 by Benali et al. [3]. In 2012, Wong et al. first generated lung organoids derived from human-induced pluripotent stem cells (IPS) as a proof of concept to model cystic fibrosis [4]. These protocols were further adapted to develop mature lung organoids that resembled proximal airways and featured upper-airway-like epithelium containing ciliated cells, basal cells and club cells, as well as mesenchymal cells such as myofibroblasts [5]. The use of human pluripotent stem cells to grow lung bud organoids in Matrigel 3D culture has further been used to model bronchiolitis following respiratory syncytial virus infection and fibrotic lung diseases in vitro [6]. The co-culture of human, adult-derived bronchial epithelium alongside an integrated stroma and lung microvascular endothelial cells was first described in 2017 by Tan et al. and demonstrates that a population of differentiated, commercially available cells carry the potential to aggregate and self-assemble towards lung tissue-like structures [7]. In the current coronavirus disease 2019 (COVID-19) pandemic, both stem-cell-based organoid models and primary alveolar cell-derived organoids have been used to model organotropism, pathogenesis and inflammatory responses following a severe acute respiratory syndrome coronavirus-2 (SARS-CoV-2) infection [8,9].

In parallel to the innovations in de novo airway tissue engineering, a protocol for a consistent three-dimensional culture of patient-derived NSCLC cells was first reported in 2013 by Endo et al. Using a serum-free human embryonic stem cell culture medium, the group achieved an 80% success rate in culturing NSCLC organoids in Matrigel [10]. In a growth assay, a comparable growth effect was seen using a medium consisting of DMEM/F12 and the HER3 ligand Neuregulin 1 [10] Upon characterization by histology and flow cytometry, the cells in NSCLC organoids were almost exclusively of epithelial origin, suggesting a pure selection of lung cancer cells using this protocol. However, no sequencing or mutational analysis was performed for further characterization and the authors did not report the duration of culture [10].

Despite these early advances in the establishment of benign airway organoids and NSCLC organoids, a protocol for long-term expansion was only described in 2019 by Sachs et al. [11]. Shortly after, several protocols for the establishment of primary, patient-derived NSCLC organoids have followed. The different NSCLC organoid systems reported to date are compared in Table 1. 

A central challenge in long-term expansion of NSCLC organoids is the early and rapid overgrowth by normal airway tissue. The lack of a selective advantage of tumor cells over normal cells is already known from organoid cultures of cancers of other origins such as colorectal and prostate cancer [19]. In colorectal cancer, the commonly encountered Wnt activation in cancer cells and the Wnt dependency of normal colonic tissue is utilized to selectively expand tumor organoids in a Wnt3a-depleted medium [19]. In prostate cancer, the common overgrowth of normal prostatic tissue only allows us to culture primary cancer organoids from metastatic lesions [20,21]. In their study, Sachs et al. used the MDM2 antagonist Nutlin-3a during early passages to eliminate normal, TP53 wild-type airway organoids and select for p53-mutated NSCLC organoids. However, this approach leads to a systematic loss of TP53 wild-type NSCLC specimens [16]. Furthermore, it may as well select and favor the growth of a subset of tumor-surrounding, p53-mutated, but histologically normal airway cells [16,22]. The growth medium used by Sachs et al. moreover applied a medium composition that made use of an activation of the Wnt/β-catenin signaling pathway (R-spondin 1), an inhibition of the TGF-β signaling pathway (Noggin and A83-01), and an inhibition of the Rho-kinase signaling pathway (ROCK inhibitor, Y-27632) (Table 1). The latter significantly increased the efficiency of primary organoid formation by inhibiting the ROCK-dependent programmed cell death after dissociation of primary cells from biopsies or resected tissue samples [23]. Using the Nutlin-3a selection, Sachs et al. reported an NSCLC organoid establishment rate of 28%. Shortly after the publication by Sachs et al., Kim et al. reported a method for an efficient generation and long-term culture (>10 passages) of NSCLC organoids with a success rate of 87%. Instead of using a Nutlin-3a selection, Kim et al. used a minimum basal medium without Wnt3a or Noggin, but with relatively high concentrations of epidermal growth factor (EGF, 50ng/ml) to select for NSCLC organoids and inhibit the growth of normal airway cells [12]. In contrast to other protocols, Kim et al. cytologically assessed the tissue quality before culturing NSCLC organoids and pre-emptively excluded 9 out of 32 samples (28.1%) due to insufficient tissue quality [12]. The early assessment of tissue quality may thus help to select promising specimens and may save lab resources. In a recent publication by Hu et al., a similar minimal medium composition was used for short-term organoid culture. The authors only used early-passage organoids for further analyses, but reported a high success rate of 79% in early lung cancer organoid establishment [17].

A further long-term organoid culture system was reported by Shi et al. based on a medium containing previously used supplements, but with added activation of the Hedgehog-pathway by using Smoothened Ligand (SAG) and activation of the Wnt signaling pathway by using the selective glycogen synthase kinase-3 inhibitor CHIR99021 (Table 1) [14]. However, no selection for NSCLC organoids was performed and contamination by normal airway organoids was common. While the group reported an initial organoid establishment rate of 88%, a long-term culture of pure NSCLC organoids was only successful in 15% of all cases [14]. Similar success rates were reported by Dijkstra et al. [16] and Yokota et al. [18] who followed the protocol described by Sachs et al. but initially abstained from a Nutlin-3a-based selection. In both studies, a remarkable discrepancy between the rates of initial organoid growth and the rates of histologically or genetically confirmed NSCLC organoid establishment was seen. In the study by Dijkstra et al., organoid growth was seen in 41% of all cases, but upon histological, immunohistochemical and genomic analysis, the majority of all organoids were normal airway organoids, and pure NSCLC organoids were only found in 17% [16]. Yokota et al. documented organoid growth in 83% of all cases, but only 7% of all organoids were validated as NSCLC [18]. A number of other medium compositions tested by Yokota et al. failed to reach comparable culture success as the airway organoid medium initially reported by Sachs et al. [18]

All NSCLC organoid systems mentioned above used a growth-factor-reduced, gelatinous extracellular matrix (Matrigel, Geltrex or Cultrex) to mimic the extracellular environment found in airway tissue. A different approach using ultra-low-attachment (ULA) plates was recently described by Herreros-Pomares et al. In 40% of all cases, an aggregation of organoids was seen at the bottom of the ULA plates and a long-term culture for more than 6 months and more than 30 passages was achieved [13]. However, the organoids were not histologically or genetically compared with the parental tumor and it remains unclear whether all organoids were pure NSCLC.

All recent publications reporting a long-term culture of NSCLC organoids share an approach that lacks serum-supplemented medium. The use of serum-free supplements such as B27 is supported by studies that show an accumulation of genetic alterations after long-term expansion of cells in serum-supplemented medium [24,25]. In addition, a serum-free culture generates a defined culture condition that increases the comparability and external validity of the findings. 

While most NSCLC organoids were established from tissue of the primary tumor, several groups reported organoids derived from extrapulmonary metastases or pleural effusion [11,16,18]. With the lack of competitive growth from normal airway tissue and a potentially more aggressive tumor biology, a higher success in organoid establishment is reported from extrapulmonary NSCLC metastases [16]. Apart from intra- or extrapulmonary tumor location, no significant associations between the establishment rate and clinicopathological variables such as tumor stage, histology, mutational status and disease-free survival were reported [13,14,16]. However, the small sample sizes currently limit further analyses and interpretations. For broad clinical applications such as personalized drug screenings, the establishment of organoids from the primary tumor, including early-stage tumors, remains essential.

Among the abovementioned culture systems, the protocols published by Sachs et al. and Kim et al. appear to offer the highest success rates. Until today, it has remained unclear whether a Nutlin-3a-based selection or other selection approaches such as a minimum basal medium offer higher establishment rates and higher tumor purity. Furthermore, the use of the same protocols has led to varying success rates among different research groups. This circumstance highlights the importance of standardization in the reporting of organoid cultures such as individual tumor cell content, and a standardized method of validating pure NSCLC organoids. 

## 3. Characterization of Primary Non-Small Cell Lung Cancer Organoids

**Figure 1:**
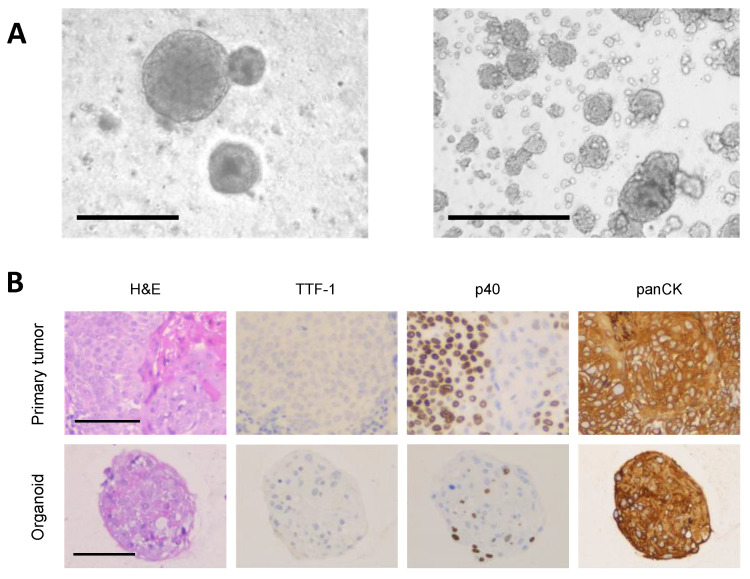
(**A**) Primary NSCLC organoids established from fresh cancer tissue. Left: lung adenocarcinoma organoids, right: lung carcinoid organoids. Scale bar: 100 μm. (**B**) Histomorphological and immunohistochemical characteristics of primary lung squamous cell carcinoma organoids and their respective parental tissue, featuring intracellular keratinization, intercellular bridges and partial p40 positivity. Scale bar: 100 μm (unpublished data, Department of Thoracic Surgery, University Hospital Zurich).

As described above, an overgrowth by normal airway organoids is commonly seen during the culture of primary NSCLC organoids. For downstream applications of the NSCLC organoids, an exact characterization is therefore central and should always be performed and reported. However, the determination of tumor purity in NSCLC organoids is challenging and several different approaches have been used in previous publications. Tumor organoids can have a variety of different appearances and cannot be differentiated from normal airway organoids by brightfield microscopy of the organoids in culture or by histomorphology (hematoxylin and eosin staining) alone (Figure 1A) [16]. Additional immunohistochemical and genetic analyses are therefore essential to determine the organoids’ origin. Immunohistochemistry (IHC) of NSCLC organoids should include the same markers that are used in the clinical diagnosis of NSCLC. For immunohistochemical differentiation between different subtypes of NSCLC, staining of thyroid transcription factor-1 (TTF-1), p40 (or p63) and a cytokeratin-based staining of epithelial cells (e.g., pan-Cytokeratin) are commonly used in clinical routine [26]. While lung adenocarcinomas are commonly TTF-1 positive, squamous cell carcinomas are generally p40 positive [26]. In order to validate and characterize primary NSCLC organoids, staining of the parental tumor and the corresponding organoids should be performed and compared (Figure 1B). Since p40 and p63 are also markers of normal lung basal cells, an expression of these markers in organoids cultured from adenocarcinoma samples may indicate normal airway tissue overgrowth and should raise doubts about the malignant origin of the organoids [14,16]. However, our own experience has shown that in rare cases, a selection of a squamous cell subpopulation in an adenosquamous carcinoma specimen can result in a similar appearance (unpublished own data). Based on morphological and immunohistochemical data, Dijkstra et al. described different phenotypes of organoids derived from intrapulmonary NSCLC: NSCLC organoids can be both cystic or solid, although most cancer organoids appear to grow as irregularly formed, solid structures [16]. In general, there appears to be no clear association between the parental tumor histology and the general NSCLC organoid morphology and architecture [17]. p63 staining is either disorganized (in squamous cell carcinoma) or absent (in adenocarcinoma), but without a clear polarization within the organoid. A TTF-1 positivity is only seen in adenocarcinoma-derived organoids. Cytomorphologic characteristics of malignancy such as nuclear and cellular pleomophism or hyperchromasy are present. In contrast, normal airway organoids most commonly show a cystic structure with a polarized p63-positive outer cellular ring and p63-negative intraluminal cells. In certain cases, normal airway organoids can also have a solid shape, but maintain the characteristic polarized p63 (or p40) staining pattern [16]. A genetic analysis helps to further characterize the origin of established organoids. Dijkstra et al. recommend copy number (CN) profiling for it is fast, affordable and does not require large amounts of organoid tissue. However, according to The Cancer Genome Atlas (TCGA), up to 15% of adenocarcinomas and 7% of squamous cell carcinomas present with a normal CN profile and are therefore difficult to distinguish from normal airway tissue based on this method [16]. Other groups used whole-exome sequencing (WES) or targeted sequencing for oncogenic drivers to verify the organoids’ malignant origin [11,12,14,15,17,18]. However, due to an inherent genetic drift during organoid passage, genetic alterations or an inconsistency in the present mutations was reported in several cases [12,18]. We therefore recommend that the characterization of primary NSCLC organoids should always include a histomorphological and immunohistochemical assessment, combined with a genetic analysis at least by CN profiling or, ideally, by WES. 

## 4. Lung Cancer Organoids as Model Systems for Lung Cancer Biology Research 

Traditional 2D cell culture models as well as transgenic mouse models are used to understand the underlying molecular mechanisms and pathway alterations leading to development and progression of cancer or to the development of drug resistance. Tumor organoids, as well as organoids generated from normal non-malignant cells, hold the potential to be used for the same purposes and to serve as ideal models for lung cancer biology research. Lo and colleagues, who have recently performed a comprehensive review of the available literature, have suggested organoids to be suitable for various cancer biology applications, including as a platform for functional genomics for oncogene discovery, to study tumor genome evolution and cancer stem cells, as well as the involvement of oncogenic pathogens [23]. Lung cancer organoids hold the potential to be used for all of these described applications. In particular, the use as a platform for understanding tumor genomic evolution could be of particular interest, in order to elucidate how under the selective pressure of a given targeted therapy resistance mechanisms develop. Being able to gain a deeper understanding of these processes might allow us to identify alternative treatment strategies for those patients developing resistance, e.g., to tyrosine kinase inhibitors (TKIs). 

## 5. Clinical Applications of NSCLC Organoid Systems: Present and Future

### 5.1. Assessment of Drug Sensitivity

Patient-derived NSCLC cell aggregates with spherical shape have already been used for chemosensitivity assays before the establishment of protocols that allowed long-term expansion of NSCLC organoids. After initial adherent culture, the group of Ruppen et al. used primary cells from three patients with NSCLC to form spherical aggregates by sedimentation in U-bottom microwells of a microfluidic device [27]. A subsequent cisplatin chemosensitivity assay in the microfluidic device demonstrated an increased chemoresistance when NSCLC cells were co-cultured with primary pericytes compared to a monoculture, suggesting a protective effect of pericytes for cancer cells [27].

To date, only few publications report the use of primary NSCLC organoids for drug screening. As an initial proof of concept, Endo et al. showed that growth of EGFR-mutated NSCLC organoids was suppressed by the EGFR TKIs erlotinib and gefitinib. These findings were further validated in PDX models [10]. Sachs et al. then demonstrated differential responses of NSCLC organoids to conventional chemotherapeutics such as cisplatin or paclitaxel, but also showed a sensitivity towards the TKIs such as erlotinib and gefitinib in ERBB2-mutant organoids [11]. In the study by Kim et al., a response to olaparib was seen in BRCA2-mutant NSCLC, a response to erlotinib in EGFR-mutant NSCLC and a response to crizotinib in EGFR-mutant and MET-amplified NSCLC organoids. The feasibility of a high-throughput drug response screening using 24 anti-cancer drugs including conventional chemotherapeutics and targeted treatments was demonstrated recently by Li et al. Notably, drug sensitivity remained consistent between different passages and drug responses correlated with the mutational profile of the parental NSCLC [15]. Shi et al. showed a strong synergetic effect using a combination treatment consisting of the MEK inhibitor trametinib and the FGFR inhibitor infigratinib in an organoid model of FGFR1 amplified lung squamous cell carcinoma [14]. Yokota et al. demonstrated that EGFR-TKI-resistant NSCLC organoids may respond to combination treatment of the Bcl-2 inhibitor navitoclax and the survivin inhibitor YM-155, and that BRAFG469A-mutated organoids were suppressed by a combination treatment of trametinib and erlotinib [18].

Despite these examples of successful drug screenings, a systematic clinical application of organoid models for personalized treatment decisions is not in sight. The time required to establish well-growing primary organoid cultures and the current rates of success and expansion limit the clinical application of organoids not only in NSCLC, but also in other cancer types such as colorectal cancer [28]. A promising solution for a faster and more straightforward drug screening is presented by Hu et al. [17] The group used early-passage (mostly p0) organoids in a microwell array chip for high-throughput analysis in a nanoliter scale and obtained drug response profiles within a week [17]. Being aware of the occurrence of genetic drift and a potential selection of rapidly growing and pluripotent cancer cells in advanced passages, the use of early-passage organoids may furthermore help to faithfully recapitulate the tumor heterogeneity and in vivo drug response and additionally fit the short time frame for an early clinical application after surgical resection [23]. Moreover, future organoid-based drug screenings should take into account biopsies from different tumor sites to help capture the parental tumor’s intratumoral heterogeneity more precisely [23].

### 5.2. Studying Cancer Stem Cells

The “cancer stem cell hypothesis” suggests that intratumoral heterogeneity is a result of the asymmetric cell division of a rare stem cell subpopulation with the capacity of self-renewal and pluripotency, which gives rise to a differentiated, but phenotypically diverse progeny [29]. In this sense, intratumoral heterogeneity and cancer stem cells are seen as the driving force behind minimal residual disease and resistance [29,30]. The investigation of cancer stem cells thus helps to understand NSCLC progression, and NSCLC stem cells are clinically promising targets for future treatment strategies. For this reason, the presence of cancer stem cell candidates in NSCLC organoids has been investigated in a small number of studies with diverging findings. Endo et al. reported no enrichment in CD133-positive cancer stem cell candidates among the organoids established in Matrigel and human embryonic stem cell culture medium. In contrast, Herreros-Pomares et al. reported an overexpression of genes related to stemness, namely p21, Notch3, CD44, integrin α6, Nanog and Snail, in lung adenocarcinoma organoids when compared to the adherently growing cells of the same tumor [13]. The group then generated a prognostic score based on the significantly overexpressed stemness genes to predict overall survival. However, no significant difference in the expression of stemness-related genes was found in lung squamous cell carcinoma [13].

### 5.3. Whole-Organoid Xenografts

In the studies by Kim et al., Shi et al. and Herreros-Pomares et al., the tumorigenic potential of organoids was assessed by xenografting whole organoids in immunodeficient mice [12,13,14]. In the xenograft, the key biological and histological properties and the tumorigenicity of the parental tumor were preserved [14]. Upon transplanting NSCLC organoids, a faster growth and higher success arte in the establishment of the xenograft tumors was seen when compared to a parallel transplantation of dissociated cells into the same animal [12,13]. 

## 6. Challenges and Perspectives

Despite the fact that numerous studies in lung cancer and other cancers have shown the promise of cancer organoids as ideal in vitro model systems for both basic and clinical cancer research, in particular in NSCLC, a number of challenges remain that need to be addressed in order to facilitate their future use, especially in direct clinical applications.

The major challenge is probably that of obtaining sufficient tissue, or more precisely a sufficient number of viable cancer cells required for a timely establishment of organoids suitable in size and number for clinical applications. In particular, drug sensitivity testing, with the aim of selecting the ideal treatment of a specific patient, requires a fast establishment of well-growing representative organoids, which can provide information regarding the response of the tumor to the various treatments with 2-4 weeks of tumor sampling, as only then does the patient truly benefit from such an approach. For such applications, tumoroids need to grow fast and reliably, but based on information from the available literature and our own experience, the time frame from surgical resection or biopsy of the cancer to the establishment of long-term cultured tumoroids, as well as establishment rates of well-characterized and pure NSCLC organoids, are currently limiting factors for the broad clinical application. Hence, available protocols will have to undergo additional optimization procedures to further develop the methodology into this direction. 

This challenge is accompanied by an important aspect we face in many areas of developing precision medicine; for example, also in the area of biomarker development, namely the standardization and validation of methodology. The various studies undertaken thus far have applied different protocols varying in almost every aspect, from sample collection procedure over tissue digestion and media composition to culture passaging conditions. However, if (lung cancer) organoids are to enter clinical applications, these processes need to be standardized, and individual steps in the process have to be performed in the same fashion in each laboratory around the world. Only then is the validation of specific approaches and verification of findings, especially in the area of the identification of novel therapeutic strategies, drug-induced molecular/genetic changes and the role of the tumor microenvironment, truly possible. A standardized characterization of all NSCLC organoids used for research purposes is crucial and we suggest a combination of histomorphologic, immunohistochemical and genetic (CN profiling or WES) assessment to confirm the malignant origin and the faithful recapitulation of the parental tumor. As described above, it is, for example, still unclear if specific selection approaches offer advantages regarding establishment rates or tumoroid purity. These questions can only be answered if they are addressed systematically by following standardized protocols that test several culture media compositions in parallel, identifying those that represent the most promising choices. A standardized protocol would allow us to generate meta-analyses that help to establish internationally recognized standard protocols. One however also has to be aware of the fact that due to the intertumoral heterogeneity of NSCLC, it is unlikely to identify a one-fits-all protocol, but through systematic evaluation it is likely to be possible to identify culture conditions most suitable for the different histological and, if the information is available, also molecular subtypes. Such standardization always requires the collaboration of the international research community, especially when dealing with a heterogenous disease, and when some of the molecular and genetic alterations one might be studying are rather rare.

## 7. Conclusions

Among the protocols published to date, only few allow us to establish patient-derived, pure NSCLC organoids with high success rates for long-term culture. The growth advantage of normal airway tissue over NSCLC tissue requires selective culture conditions that may lead to a systematic loss of certain NSCLC subtypes. Further advancements in culture protocols are therefore required before NSCLC organoids can be broadly used for clinical applications, such as personalized treatment decisions on the basis of drug screenings. A standardized, parallel and prospective assessment of different culture protocols conducted in different research centers may help to identify potential phenotype- or genotype-specific supplements that enhance organoid growth and advance the methodology for clinical applications. For drug screening, early-passage organoids may be best suited, since they recapitulate the parental tumor most closely and provide results within a short time span.

## Figures and Tables

**Table 1:** Primary non-small cell lung cancer organoid publications.

Author	Year	Medium	Supplements	ECM	Method	Success Rate	*n* (Establ.)	Validation	Downstream Applications Tested
Endo et al. [10]	2013	StemPro hESC medium *or* DMEM/F12, Bovine serum albumin and Neuregulin 1	n/a	Matrigel (GFR)	Neuregulin 1	80%	100	Histology and IHC Flow cytometry	Response to erlotinib
Sachs et al. [11]	2019	Advanced DMEM/F12	R-Spondin 1 FGF7, FGF10, Noggin A83-01 ROCK Inhibitor SB202190 B27 Nutlin-3a	Cultrex Basal Membrane Extract (GFR)	Nutlin-3a selection	28%	18	Histology and IHC Whole-genome sequencing	Drug response including targeted therapy
Kim et al. [12]	2019	DMEM/F12	Basic FGF Human EGF ROCK Inhibitor N2 B27	Matrigel (GFR)	Minimum basal medium	87%	20	Histology and IHC SNP genotyping, targeted sequencing, whole-exome sequencing	Drug response including TKIs and PARP inhibitor Whole-organoid xenografts
Herreros-Pomares et al. [13]	2019	Advanced DMEM/F12	Basic FGF Human EGF Bovine serum albumin	n/a	Ultra low attachment plates	40%	8	Microscopy	Cytotoxicity assays Whole-organoid xenografts Prognostic score for overall survival
Shi et al. [14]	2020	Advanced DMEM/F12	Human EGF FGF4, FGF10 Noggin A83-01 ROCK Inhibitor B27 CHIR99021 Smoothened Ligand (SAG)	Matrigel (GFR)	M26 medium	88% organoid establishment 15% non-contaminated long-term culture	47 (short-term) 10 (long-term)	Histology and IHC Whole-exome sequencing and CNV analysis RNA-seq	Targeted therapy response including combination treatments Whole-organoid xenografts
Li et al. [15]	2020	Advanced DMEM/F12	R-Spondin 1 FGF7, FGF10, Noggin A83-01 ROCK Inhibitor SB202190 B27	Matrigel (GFR)	no selection	n/a	12	Histology and IHC Whole-exome sequencing RNA-seq	High-throughput drug dose–response screens
Dijkstra et al. [16]	2020	Advanced DMEM/F12	R-Spondin 1 FGF7, FGF10, Noggin A83-01 ROCK Inhibitor SB202190 B27 Nutlin-3a	Geltrex Basement Membrane (GFR)	no selection	17%	10	Histology and IHC Copy number profiling	n/a
Hu et al. [17]	2021	DMEM/F12	Human EGF ROCK Inhibitor N2 B27 SB202190 A83-01 Forskolin	Matrigel (GFR)	Minimum medium	79%	77	Histology and IHC Whole-exome sequencing RNA-seq	Early-passage drug response profiling on microwell arrays
Yokota et al. [18]	2021	Advanced DMEM/F12	R-Spondin 1 FGF7, FGF10, Noggin A83-01 ROCK Inhibitor SB202190 B27 Nutlin-3a	Cultrex Basal Membrane Extract (GFR)	Nutlin-3a selection	7%	3	Histology and IHC Sanger Sequencing Karyotyoing	Drug response including targeted therapy

Additional antibiotic agents, antioxidants, co-enzymes, trace elements and buffers may vary. n/a, not available.
